# Predictors of Sleep Quality in Spouse Caregivers of Community-Dwelling People With Dementia Using Propensity Score Matching Analysis

**DOI:** 10.1097/jnr.0000000000000582

**Published:** 2023-11-13

**Authors:** Hyeon Sik CHU, Hye-Young JANG

**Affiliations:** 1 PhD, RN, Assistant Professor, College of Nursing, Dankook University, Republic of Korea; 2 PhD, RN, Associate Professor, College of Nursing, Hanyang University, Seoul, Republic of Korea.

**Keywords:** dementia, spousal primary caregiver, Korea, propensity scores, sleep quality

## Abstract

**Background:**

Many family caregivers of people with dementia (PwDs) have sleep problems and poor sleep quality. Sleep may be negatively affected by caring for a family member with dementia, especially a spouse.

**Purpose:**

This study was designed to assess sleep quality in spouse caregivers of PwDs and determine the impact of care provision on their sleep quality.

**Methods:**

A secondary analysis of 58,050 participants in the 2018 Korea Community Health Survey was conducted. To prevent selection bias, a propensity score matching analysis was performed. Multiple logistic regression analysis was conducted to investigate the predictors of sleep quality.

**Results:**

After obtaining a propensity score matching threshold of 3:1, the percentage of poor sleepers was 24.2% in the control group and 33.3% in the spouse-caregiver group, which indicates a significant difference (χ^2^ = 11.79, *p* = .001). After adjusting for depressive symptoms in the multiple logistic analyses, no intergroup difference was found in terms of risk of poor sleep quality (odds ratio = 1.12, 95% CI [0.90, 1.61]).

**Conclusions/Implications for Practice:**

The findings of this study support that spouse caregivers of PwDs have poorer sleep quality than their nonspouse peers and that management of depressive symptoms is important to improving the sleep quality of spouses providing care to PwDs. Nursing interventions such as light therapy and exposure to sunlight during daytime hours to both improve sleep quality and reduce depressive symptoms can improve sleep quality in this vulnerable caregiver group.

## Introduction

The growing older adult population worldwide is increasing the prevalence of dementia. The number of people with dementia (PwDs) globally is projected to increase from 50 million in 2020 to 82 million in 2030 and 152 million in 2050 (Alzheimer's Disease International et al., 2020). In Korea, the number of PwDs among adults aged ≥ 65 years in 2020 was 840,000 and is expected to exceed three million by 2050 (Ministry of Health and Welfare & Central Dementia Center, 2022). Dementia is a neurodegenerative disease associated with various problems, including cognitive decline, speech dysfunction, and behavioral and psychological symptoms (Cerejeira et al., 2012; Sato et al., 2018), that make it difficult for PwDs to perform independent activities of daily living and necessitate continuous nursing care (Khanassov et al., 2021; Prince et al., 2016). In general, family members are the primary source of care for PwDs, with many PwDs cared for at home (Andreakou et al., 2016).

In Korea, PwDs are generally cared for by their adult children. However, the number of spouses taking care of PwDs has been rising because of the increasing prevalence of nuclear families and other changes to the traditional family structure (Jang & Yi, 2017; Park et al., 2015). The percentage of spouses serving as family caregivers rose from 37.7% to 56.6% between 2014 and 2019 and is expected to rise further (Statistics Korea, 2019). Considering these changes in the reality of support, having a multifaceted understanding of spousal caregiving is of the utmost importance.

Providing care to a PwD significantly affects caregivers in multiple dimensions, including physical, psychological, social, and economic. Poor sleep quality decreases immune function and overall quality of life, especially in spouses serving as primary caregivers. Considering that these spouses are also older individuals, their experience caring for PwDs is inherently different from that of caregivers who are the adult children of PwDs.

A study on the caregiving experience that considered caregiver characteristics found role burden, psychological distress, economic issues, and health problems to be higher (Park et al., 2015) and health-related quality of life to be lower (Kim & Yeo, 2012) in spouses than in adult children. This situation is limited not only to the difficulties faced by caregivers but also to the quality of life of PwDs. Therefore, active, effective, and differentiated support measures that consider the characteristics of spouse caregivers are required.

Sleep quality is one of the health issues experienced by family caregivers of PwDs (Polenick et al., 2018; Wilson et al., 2019). Sleep disturbances have been found in two thirds of family caregivers of PwDs (Liang et al., 2020; Peng et al., 2019). In particular, spouse primary caregivers tend to experience greater sleep disturbance because they not only live with PwDs but also sleep beside them (Gao et al., 2019). These sleep disturbances may be further exacerbated by psychosocial factors such as caregiving-related burden, depression, and anxiety, which worsen sleep quality (Peng et al., 2019; Smyth et al., 2020). Furthermore, old age, fatigue, and chronic diseases have also been reported to affect sleep quality among family caregivers of PwDs (Chiu et al., 2014; Smyth et al., 2020; von Känel et al., 2012).

In particular, if changes in sleep patterns because of aging are not adequately managed, sleep disorders may occur. This issue is further complicated by the fact that older adults rarely perceive or recognize sleep disturbance as a problem. Moreover, as they lack sufficient temporal and economic resources for self-care, these older adults may neglect their own health because of the immediate care needs of the PwD under their care (Simpson & Carter, 2013). Consequently, caregivers of PwDs are at a higher risk of experiencing poor sleep quality (Peng et al., 2019).

Poor sleep quality is associated with decreased immune function and increased risk of chronic diseases such as hypertension, diabetes (Hoyt et al., 2021; von Känel et al., 2006, 2010), cognitive decline (Surani et al., 2015), poor quality of life (Lippe et al., 2021), and early institutionalization in long-term care facilities (Chang & Schneider, 2010). Diabetes and hypertension are associated with reduced sleep quality because of metabolic syndrome, nocturnal hypoglycemia, peripheral neuropathy, and sleep apnea (Shantsila et al., 2021). Moreover, cognitive decline is closely related to increased stress hormone cortisol and amyloid deposition because of poor sleep quality (Joo et al., 2021).

On the basis of the above, an assessment of the relevant factors of sleep quality among family caregivers of PwDs is necessary to facilitate effective early intervention and preventive actions. Therefore, this study was designed to assess sleep quality in spouses caring for PwDs and to determine the effect of care provision on their sleep quality. This study hypothesized that (a) sleep quality in spouses of PwDs is poorer than that in spouses of patients without dementia and (b) caregiving affects sleep quality even after adjusting for health status.

## Methods

### Study Design

This secondary analysis was conducted using a cross-sectional study design. Data for this study were obtained from the 2018 Korean Community Health Survey (KCHS), a nationwide health survey conducted by the Korea Disease Control and Prevention Agency that provides population-based statistics for constructing and assessing national healthcare plans (Joo et al., 2021; Kang et al., 2015). The KCHS was performed by trained interviewers who visited the selected sample households and conducted computer-assisted individual interviews with adults aged 19 years or older.

### Participants

**Figure 1.**
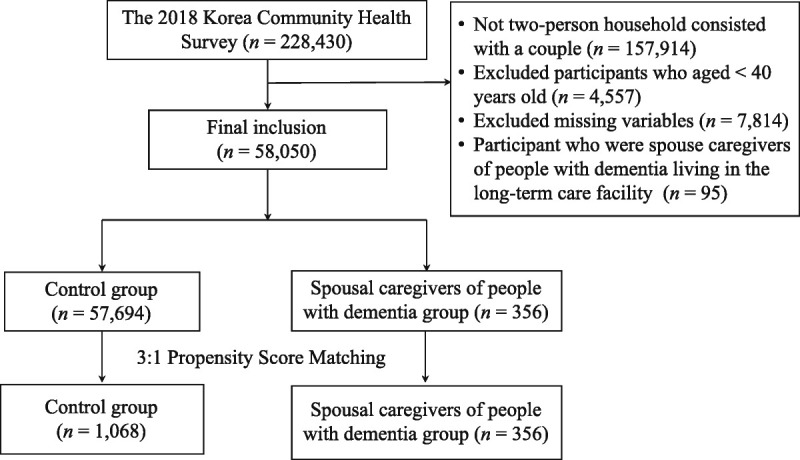
Flowchart of Participant's Selection

The participants in this study were adults aged 40 years and older living with their spouses. The spouse caregiver of a PwD was defined as an individual living with and providing care to a PwD as a spouse. The exclusion criteria were spouse caregivers caring for presenile dementia and spouses of PwDs living in long-term care facilities. The control group included individuals who were living with spouses who did not have a diagnosis of dementia. The details of the participant selection procedure are shown in Figure 1.

### Ethical Considerations

This study was approved by the institutional review board (IRB No. HYUIRB-202108-014). The KCHS raw data were obtained from a publicly available database, which is freely accessible online at http://chs.cdc.go.kr. The KCHS raw data, in accordance with the Korean Personal Information Protection Act and Statistics Act, do not include personal information or identifiers.

### Measures

#### Propensity score matching covariates

Seven sociodemographic and health-related characteristics were selected as variables for propensity score matching (PSM) for the spouse caregivers in the PwD and control groups. For sociodemographic variables, the selected variables were age, gender, educational level, monthly household income, and employment. For health-related characteristics, the selected variables were diabetes mellitus and hypertension. As these variables are not modifiable through nursing care or nursing interventions, they were controlled statistically using PSM.

#### Health-related variables

Variables adopted in this study to represent the health-related characteristics of participants were depressive symptoms, obesity, smoking, alcohol intake, self-rated health status, and subjective cognitive decline.

Depressive symptoms were measured using the Korean version of the Patient Health Questionnaire-9 (PHQ-9), a self-report scale for screening depression based on *Diagnostic and Statistical Manual of Mental Disorders* (4th ed.) criteria. Respondents are asked to rate each item on the PHQ-9 using a 4-point Likert-type scale (0–3), with total possible scores ranging from 0 to 27 and higher scores indicating more severe depressive symptoms. The PHQ-9 has been validated for use in older Korean adults, and a total score of 5 has been suggested as the optimal cutoff for screening for clinical depression (Han et al., 2008). In this study, the Cronbach's alpha for the PHQ-9 was .93. The participants were classified as either obese or nonobese (≥ 25 and <24.9 kg/m^2^, respectively) based on the body mass index criterion published by the World Health Organization Western Pacific Regional Office (Lim et al., 2017). In addition, the participants were classified based on smoking status (smoker or nonsmoker), alcohol intake (less than once a month or more than once a month), and self-rated health status (poor, fair, or good).

Subjective cognitive decline was defined as the respondent either perceiving worsening cognitive functions or experiencing increased frequency of confusion or memory problems during the past 12 months (Jessen et al., 2014). In this study, subjective cognitive decline was determined based on the answer to the following question: “Have you experienced more frequent or severe disorientation or memory loss during the last year?” The allowed responses were “yes” and “no” only (Joo et al., 2021).

#### Sleep quality

Sleep quality was measured using the Korean version of the Pittsburgh Sleep Quality Index (PSQI), which assesses sleep quality and patterns over a 1-month period (Sohn et al., 2012). The PSQI has been widely used in population-based and clinical studies and consists of 19 items and the following seven components: subjective sleep quality, sleep latency, sleep duration, habitual sleep efficiency, sleep disturbances, use of sleep medications, and daytime dysfunction. Each component may be scored from 0 to 3, and the global score for overall sleep quality is calculated by summing all of the components for a total possible index score ranging from 0 to 21. PSQI global scores > 8.5 indicate poor sleep quality in Korean populations, and this cutoff score was adopted in this study (Sohn et al., 2012). The Cronbach's alpha for the PSQI in this study was .87.

### Data Analysis

Statistical analyses were conducted using SPSS 23.0 (IBM Inc., Armonk, NY, USA) and the R program (www.r-project.org/). To avoid selection bias because of confounding covariates, the 1:3 nearest neighbor PSM with a caliper value set at 0.1 was performed using the R program (Ho et al., 2011). The 1:1 matching strategy has a disadvantage in that if the propensity scores of the control group and the retreatment groups are dissimilar, the data of a large number of treatment groups are eliminated. Therefore, 1:*n* (treatment group to control group) matching has better power than 1:1 matching in PSM. However, the increase in power is insignificant if matching is performed with 1:5 or more (Rosenbaum, 2020). This problem can be compensated for by adequately setting the caliper width. Therefore, we set the PSM ratio as 1:3 (spouse caregivers of the PwD group to nonspouse caregivers of PwDs) with a caliper width of 0.1. In studies using nearest-neighbor matching with the fixed caliper width method in the medical and nursing science field, the caliper range varied from 0.01 to 0.6 (Austin, 2009). Therefore, we set the caliper width as 0.1 and performed calculated propensity without replacement.

Multivariate logistic regression analysis was performed to identify the effects of caregiving on the sleep quality of the caregivers. A two-tailed *p* < .05 was considered to be statistically significant.

## Results

### Participant Characteristics From Unadjusted Data and Propensity Score-Matched Data

The sociodemographic and health-related characteristics of the participants according to caregiving provisions are shown in Table 1. The spouse caregivers of the PwD (experimental) group was relatively older and less educated, had more unemployed members, and had a lower average household income than the spouse caregivers of people without dementia (control) group (*p* < .001). Furthermore, the spouse caregivers of the PwD group experienced diabetes mellitus and hypertension more frequently (*p* < .001). Many differences in distribution patterns between the two groups were apparent before PSM. After PSM, similar distribution patterns appeared, and no significant intergroup differences in terms of covariates were observed, thus confirming the appropriateness of using this matching approach.

**Table 1.** Between-Group Comparison of Baseline Characteristics, Pre- and Post-Propensity Score Matching

Variable	Unadjusted Data					Propensity Score-Matched Data				
	Control (*n* = 57,694)		Spouse Caregivers of PwDs (*n* = 356)		*p*	Control (*n* = 1,068)		Spouse Caregivers of PwDs (*n* = 356)		*p*
	*n*	%	*n*	%		*n*	%	*n*	%	
Age (years; *M* and *SD*)	66.0	9.8	76.5	7.4	< .001	76.2	7.0	76.5	7.4	.571
Gender					.794					.878
Male	28,690	47.7	180	50.6		536	50.2	180	50.6	
Female	29,004	50.3	176	49.4		532	49.8	176	49.4	
Educational level					< .001					.762
≤ Primary school	23,741	41.1	241	67.7		740	69.3	241	67.7	
Middle school	10,896	18.9	59	16.6		172	16.1	59	16.6	
High school	15,132	26.2	39	11.0		118	11.0	39	11.0	
≥ College	7,925	13.7	17	4.8		38	3.6	17	4.8	
Monthly household income (USD; *M* and *SD*)					< .001					.565
< 1,000	12,458	21.6	184	51.7		565	52.9	184	51.7	
1,000–1,990	16,335	28.3	122	34.3		380	35.6	122	34.3	
2,000–2,990	11,080	19.2	30	8.4		80	7.5	30	8.4	
≥ 3,000	17,821	30.9	20	5.6		43	4.0	20	5.6	
Employment					< .001					.810
Employed	32,988	57.2	100	28.1		291	27.2	100	28.1	
Unemployed	24,706	42.8	256	71.9		777	72.8	256	71.9	
Diabetes mellitus					< .001					1.000
No	48,136	83.4	268	75.3		804	75.3	268	75.3	
Yes	9,558	16.6	88	24.7		264	24.7	88	24.7	
Hypertension					< .001					.926
No	33,479	58.0	158	44.4		469	43.9	158	44.4	
Yes	24,215	42.0	198	55.6		599	56.1	198	55.6	

*Note*. PwDs = people with dementia; USD = U.S. dollar.

### Comparison of Health Status Between the Two Groups After Propensity Score Matching

The post-PSM health-related characteristics of the control and experimental groups are presented in Table 2. Obesity, smoking, and alcohol intake were not significantly differences between the two groups. However, when the total score on the PHQ-9 was classified using a cutoff score of 5 points, the depression rate was 20.7% in the control group and 35.1% in the spouse caregivers of the PwD (experimental) group, representing a significant difference (χ^2^ = 30.18, *p* < .001). Furthermore, there were more instances of poor self-rated health status in the experimental group (χ^2^ = 9.13, *p* = .010), and the rate of subjective cognitive decline was 33.2% in the control group and 47.2% in the experimental group, representing a significant difference (χ^2^ = 22.36, *p* < .001).

**Table 2.** Between-Group Comparison of Health Status and Health Behaviors, Post-Propensity Score Matching (PSM; *N* = 1,424)

Variable	After PSM					
	Control (*n* = 1,068)		Spouse Caregivers of PwDs (*n* = 356)		χ^2^	*p*
	*n*	%	*n*	%		
Depressive symptoms					30.18	< .001
Not depressed	847	79.3	231	64.9		
Depressed (PHQ-9 ≥ 5)	221	20.7	125	35.1		
Obesity					3.46	.068
Nonobese	705	66.0	254	71.3		
Obese (BMI ≥ 25)	363	43.0	102	28.7		
Smoking					0.08	.784
Smoker	92	8.6	29	8.1		
Nonsmoker	976	91.4	327	91.9		
Alcohol intake					0.96	.347
Less than once a month	813	76.1	280	78.7		
More than once a month	255	23.9	76	21.3		
Self-rated health status					9.13	.010
Poor	468	43.9	188	52.8		
Fair	386	36.1	113	31.7		
Good	214	20.0	55	15.4		
Subjective cognitive decline					22.36	< .001
No	713	66.8	188	52.8		
Yes	355	33.2	168	47.2		

*Note*. PwDs = people with dementia; PHQ-9 = Patient Health Questionnaire-9; BMI = body mass index.

### Comparison of Sleep Quality Between the Two Groups After Propensity Score Matching

The PSQI global score was 6.24 (*SD* = 3.68) for the control group and 7.03 (*SD* = 4.00) for the experimental group, which was significantly higher. When the PSQI global score was 8.5 points, the poor sleeper rate was 24.2% for the control group and significantly higher for the experimental group (33.4%; χ^2^ = 11.79, *p* = .001). There were significant intergroup differences in PSQI dimension scores for subjective sleep quality (χ^2^ = 9.85, *p* = .020), sleep latency (χ^2^ = 14.64, *p* = .002), habitual sleep efficiency (χ^2^ = 11.19, *p* = .011), use of sleep medication (χ^2^ = 12.05, *p* = .007), and daytime dysfunction (χ^2^ = 29.93, *p* < .001; as shown in Table 3).

**Table 3.** Between-Group Comparison of Sleep Quality (*N* = 1,424)

Variable	Control (*n* = 1,068)		Spouse Caregivers of PwDs (*n* = 356)		χ^2^ or *t*	*p*
	*n*	%	*n*	%		
**Global PSQI score** (*M* and *SD*)	6.24	3.68	7.03	4.00	3.27	.001
**Good sleeper**	810	75.8	258	66.6	11.79	.001
**Poor sleeper** (PSQI > 8.5)	237	24.2	119	33.4		
PSQI subdomain						
Subjective sleep quality					9.85	.020
0 (very good)	143	13.4	30	8.4		
1 (fairly good)	611	57.2	199	55.9		
2 (fairy bad)	257	24.1	99	27.8		
3 (very bad)	57	5.3	28	7.9		
Sleep latency (score)					14.64	.002
0 (0)	386	36.1	135	37.9		
1 (1–2)	344	32.2	79	22.2		
2 (3–4)	184	17.2	78	21.9		
3 (5–6)	154	14.4	64	18.0		
Sleep duration (hours)					2.33	.508
0 (> 7)	247	23.1	87	24.4		
1 (6–7)	270	25.3	98	27.5		
2 (5–5.9)	259	24.3	73	20.5		
3 (< 5)	292	27.3	98	27.5		
Habitual sleep efficiency (%)					11.19	.011
0 (≥ 85)	670	62.7	212	59.6		
1 (75–84)	189	17.7	47	13.2		
2 (65–74)	102	9.6	45	12.6		
3 (≤ 64)	107	10.0	52	14.6		
Sleep disturbances (score)					3.96	.266
0 (0)	77	7.2	30	8.4		
1 (1–9)	837	78.4	264	74.2		
2 (10–18)	149	14.0	58	16.3		
3 (19–27)	5	0.5	4	1.1		
Use of sleep medication					12.05	.007
0 (not during the past month)	985	92.2	310	87.1		
1 (< 1/week)	19	1.8	5	1.4		
2 (1–2/week)	11	1.0	7	2.0		
3 (≥ 3/week)	53	5.0	34	9.6		
Daytime dysfunction (score)					29.93	< .001
0 (0)	780	73.0	221	62.1		
1 (1–2)	180	16.9	69	19.4		
2 (3–4)	92	8.6	45	12.6		
3 (5–6)	16	1.5	21	5.9		

*Note*. PwDs = people with dementia; PSQI = Pittsburgh Sleep Quality Index.

### Effects of Care Provision on Sleep Quality in Spouse Caregivers

The risk of poor sleep quality was higher in the experimental group than the control group after adjusting for self-rated health status in Model 1, with the risk of poor sleep quality significantly higher for spouse caregivers (odds ratio [*OR*] = 1.46, 95% CI [1.11, 1.91]). In Model 2, which is a variation of Model 1 that further adjusts for subjective cognitive decline, risk of poor sleep quality was significantly higher in the experimental group (*OR* = 1.40, 95% CI [1.07, 1.84]). In Model 3, which is a variation of Model 2 that further adjusts for depressive symptoms, risk of poor sleep quality was similar between the two groups (*OR* = 1.12, 95% CI [0.90, 1.61]; see Table 4).

**Table 4.** Multivariate Logistic Regression Models of Caregiving on Sleep Quality in Spouse Caregivers of People With Dementia (*N* = 1,424)

Variable	Crude		Model 1		Model 2		Model 3	
	*OR*	95% CI	*OR*	95% CI	*OR*	95% CI	*OR*	95% CI
Caregiving								
Control (reference)			1		1		1	
Spouse caregiver	1.58*	[1.21, 2.05]	1.46*	[1.11, 1.91]	1.40*	[1.07, 1.84]	1.12	[0.90, 1.61]
Self-rated health status								
Good (reference)			1		1		1	
Fair			1.50	[0.98, 2.29]	1.50	[0.98, 2.29]	1.32	[1.65, 3.76]
Bad			4.01**	[2.72, 5.93]	3.34**	[2.60, 5.68]	2.49**	[0.85, 2.04]
Subjective cognitive decline								
No (reference)					1		1	
Yes					1.36*	[1.06, 1.75]	1.00	[0.76, 1.32]
Depressive symptoms								
Not depressed							1	
Depressed							1 5.25**	[3.94, 6.98]

*Note*. *OR* = odds ratio; CI = confidence interval.

Hosmer-Lemeshow goodness of fit statistic (χ^2^ = 7.972, *p* = .335); Negelkerke *R*^2^ = .242; Cox and Snell *R*^2^ = .166.

*p < .05. **p < .001.

## Discussion

In this study, sleep quality was compared between spouse caregivers of PwDs and spouse caregivers of people without dementia using PSM to reduce data heterogeneity and randomize allocation. In addition, we investigated the effect of caregiving on depressive symptoms among spouse caregivers of PwDs even after adjusting for health status.

Comparing the health status and health-related behaviors that can affect depressive symptoms and sleep quality of the two groups showed the experimental (spouse caregivers of PwDs) group had significantly lower self-rated health status than the control group. This result aligns with those of previous studies that found a negative impact of caregiving on spouse caregivers (Brown & Cohen, 2020). Negative physiological responses such as the dysregulation of the hypothalamic–pituitary–adrenal axis may lead to subjective cognitive decline in spouse caregivers of PwDs (Oken et al., 2011). Moreover, self-rated health status may deteriorate because of chronic stress (Hoyt et al., 2021; Mallya & Fiocco, 2018).

In addition, obesity, smoking, and alcohol intake did not differ significantly between the control and spouse caregiver groups. This result contradicts the findings of previous studies, which reported caregiving-related stress and lifestyle changes increase the risks of obesity and smoking and alcohol consumption among spouse caregivers (Carpenter et al., 2020; Gottschalk et al., 2020). Thus, we speculate that these descriptors are correlated but not causally related. In this study, there were no significant differences identified between the two groups in terms of obesity, smoking, or alcohol intake because the sociodemographic variables that may most strongly influence health risk behaviors were controlled using PSM.

In the stress process model of family caregiving (Pearlin et al., 1990), the underlying premise of the conceptual scheme is that one set of stressors leads to another set of stressors. In other words, the primary stress caused by continuous care provision causes secondary stress consisting of various intrapsychic strains. Therefore, in this study, self-rated health status in the experimental group was lower than that in the control group because of the stress of care provision, which may have affected depressive symptoms and physical health, leading to poorer sleep quality (Judge et al., 2010).

The results of this study concur with those of Gao et al. (2019), which identified sleep quality and sleep time in family caregivers of PwDs as significantly lower than in age-controlled noncaregivers. In particular, family caregivers of PwDs exhibited difficulties falling asleep, poor habitual sleep efficiency, and sleep disturbances. In addition to PwD-related factors such as health status and cognitive functions, family caregiver factors, including depressive symptoms, fatigue, and anxiety, were also shown to relate to sleep quality (Byun et al., 2016).

Multivariate logistic regression analysis was performed in this study to confirm the risk of poor sleep quality. After adjusting for self-rated health status and subjective cognitive decline, the risk of poor sleep quality was 1.40 times higher in the experimental group than the control group. However, the difference was not significant after adjusting for depressive symptoms, indicating that depressive symptoms contribute significantly to sleep quality in spouse caregivers of PwDs. Taking care of PwDs at home is burdensome, and spouse caregivers who stay at home with PwD experience high levels of stress (Joling et al., 2015). In addition, behavioral psychological symptoms in PwDs such as wandering, nighttime behaviors, and sunset syndrome have been associated with depressive symptoms in spouse caregivers (Byun et al., 2016) and identified as affecting sleep quality because of their influence on the circadian rhythm (Gao et al., 2019; McCurry et al., 2007). Therefore, it is important to consider the effect of depressive symptoms on sleep quality in spousal caregivers.

Previous observational studies of depressive symptoms and sleep quality in caregivers of PwDs used nonrandom sampling methods, which introduced the potential of uncontrolled selection bias. Selection bias, which reflects intergroup heterogeneity, may lead to incorrect inferences in terms of overestimating or underestimating the errors and the results. Thus, this study is notable in its utilization of PSM analysis to control for confounding variables and to reduce selection bias.

This study was affected by several limitations. Because this was a secondary analysis of primary data from the 2018 KCHS, variables related to the characteristics of PwDs that may affect quality of sleep in spouse caregivers were not included as covariates. In addition, the cross-sectional design approach used prevented us from making causal inferences about the directionality of the identified relationships. Furthermore, the lack of objective measures of sleep quality available for consideration in this study precluded our verification of the accuracy of the data provided in the self-report questionnaires.

This study has several important implications. First, because quality of sleep affects both physical and mental health, maintaining good health status is important to maintaining quality of life for both family caregivers of PwDs and PwDs receiving care at home. Therefore, healthcare professionals should regularly assess depressive symptoms in these caregivers and their quality of sleep. Moreover, to qualitatively improve their sleep quality, spouse caregivers of PwDs should receive interventions, including education related to sleep hygiene, sleep environment modification, and light chronotherapy (Fernández-Puerta et al., 2022), to improve their depressive symptoms and related behaviors.
